# Diverse responses of hypoxia-inducible factor alpha mRNA abundance in fish exposed to low oxygen: the importance of reporting methods

**DOI:** 10.3389/fphys.2024.1496226

**Published:** 2024-10-04

**Authors:** Taylor E. Murphy, Bernard B. Rees

**Affiliations:** Department of Biological Sciences, University of New Orleans, New Orleans, LA, United States

**Keywords:** oxygen, gene expression, transcription factor, hypoxia inducible factor, quantitative PCR

## Abstract

Low dissolved oxygen (hypoxia) poses significant challenges to aquatic ecosystems, affecting the behavior, reproduction, and survival of aquatic organisms. Some fishes respond to hypoxia by changes in gene expression, which may be regulated by the hypoxia inducible factor (HIF) family of transcription factors. HIF abundance and activity depends upon the post-translational modification of the alpha protein subunit, although several studies indicate that *HIFA* mRNA abundance increases in tissues of fishes exposed to hypoxia. This study reviewed reports of laboratory exposures of adult ray-finned fishes to hypoxia and used generalized linear mixed effects models to examine the influence of *HIFA* gene, tissue sampled, and exposure conditions in explaining the diversity of responses seen in *HIFA* mRNA abundance. The frequency of hypoxia-induced increases in *HIFA* mRNA was poorly explained by gene, tissue, or the severity of the hypoxic exposure. Rather, the frequency of reported increases was strongly related to the extent to which studies adhered to guidelines for documenting quantitative real-time PCR methods: the frequency of hypoxia-induced increases in *HIFA* mRNA decreased sharply in studies with more thorough description of experimental design. Future research should (a) adhere to stringent reporting of experimental design, (b) address the relative paucity of data on *HIF2A* and *HIF3A*, and (c) determine levels of HIF alpha protein subunits. By following these recommendations, it is hoped that a more complete understanding will be gained of the role of the HIF family of transcription factors in the response of fish to hypoxia.

## 1 Introduction

Recent decades have seen an increase in the frequency and severity of aquatic hypoxia on a global scale (Breitburg et al., 2018), and the ability of fish to survive hypoxia requires a range of behavioral, physiological, and biochemical strategies (Chapman and McKenzie, 2009; Richards et al., 2009). Because many of these responses may depend on changes in gene expression (Nikinmaa and Rees, 2005; Shen et al., 2023), there is considerable interest in understanding the control of gene expression in fishes during exposure to low oxygen. The hypoxia-inducible transcription factors (HIFs) are evolutionary conserved central regulators of the molecular responses of animals to low oxygen (Semenza, 2012; Pamenter et al., 2020; Mandic et al., 2021). The active transcription factor is comprised of an alpha subunit (HIFα) and a beta subunit (HIFβ) (Semenza, 2012). HIFβ, also known as the aryl hydrocarbon receptor nuclear translocator (McIntosh et al., 2010), is constitutively expressed, whereas the cellular abundance and activity of HIFα are oxygen-dependent. At normal oxygen levels (normoxia), hydroxylation of specific proline residues targets HIFα for rapid proteasomal destruction (Semenza, 2012). In hypoxic conditions, however, the degradation of HIFα is blocked, leading to its accumulation. It then dimerizes with HIFβ, translocates into the nucleus, and activates gene expression (Semenza, 2012).

Signaling by HIF has been best studied in mammals, which possess three alpha subunits, HIF1α, HIF2α, and HIF3α, encoded by genes, *HIF1A*, *HIF2A*, and *HIF3A*, respectively (Pamenter et al., 2020). The initial phylogenetic analysis of *HIFA* genes in ray-finned fishes (Actinopterygii) demonstrated that they possess orthologs of the three genes found in mammals (Rytkonen et al., 2011). Subsequently, Rytokonen et al. (2013) showed that the cyprinids (carp and related species, including zebrafish and goldfish) possess teleost-specific duplicates, *HIF1Aa/b*, *HIF2Aa/b*, and *HIF3Aa/b*. Townley et al. (2022) supported the presence of *HIF1Aa/b* and *HIF2Aa/b* in a larger clade of fishes (Otocephala), which includes not only cyprinids, but also catfish, tetras, and herring. In addition, Townley et al. (2022) presented evidence that *HIF3Aa* and *HIF3Ab* correspond to one copy each of *HIF3A* and *HIF4A* in ray-finned fishes, which resulted from the genome duplication events at the base of vertebrate evolution. Moreover, Townley et al. (2022) showed that Salmonidae (salmon and trout) have independently evolved duplicates of *HIF1Aa*, *HIF2Aa*, and *HIF3A*. Thus, bony fishes display a diversity of *HIFA* genes that far exceeds the described diversity in other vertebrate lineages, demanding special attention when differentiating among them.

In the first report of HIF from fish, Soitamo et al. (2001) showed that HIF1α protein increases in abundance during hypoxic exposures of fish cells in culture in the absence of any change in *HIF1A* mRNA abundance, and that treatment of normoxic cells with an inhibitor of the proteasome recapitulated this increase. These results suggested that protein stabilization, rather than new transcription, was the mechanism of HIF1α upregulation in fish as it is in mammals. Since then, however, several studies have reported elevated mRNA levels of various *HIFA* genes in tissues of fish exposed to low oxygen (Mandic et al., 2021). In this mini-review, we attempt to discern if there are common patterns of *HIFA* mRNA responses to low oxygen among fishes. Specifically, we ask if the variation in reported increases in *HIFA* mRNA levels during hypoxia is related to the *HIFA* gene measured, tissue sampled, severity of hypoxia, or other experimental details (Bustin et al., 2009).

## 2 Methods

One hundred and forty articles were identified through searches in Web of Science using the following terms: “HIF (Topic) and mRNA (All fields) and fish (All fields)” or “hypoxia-inducible factor (Topic) and mRNA (All fields) and fish (All fields).” These articles were filtered to remove those on organisms other than ray-finned fishes (35 studies), or early developmental stages (embryos and larvae; 42 studies). The dataset was further filtered to include only studies using laboratory exposures with defined conditions (DO levels and exposure durations), that reported real-time qPCR data with associated statistical analyses. The final dataset consisted of 38 studies reporting on *HIFA* mRNA abundance in tissues of 25 species of ray-finned fishes.

We extracted information on *HIFA* gene(s) analyzed, tissues sampled, DO concentration, duration of exposure, temperature, and salinity (Supplementary Data Sheet S1). The identity of each *HIFA* gene was verified by searching author-reported accession numbers or primer sequences against all ray-finned fishes using Basic Local Alignment Search Tool (Sayers et al., 2022). We follow the nomenclature of Townley et al. (2022) in distinguishing among *HIF1A*, *HIF2A*, *HIF3A*, and *HIF4A*, as well as between teleost-specific duplicates *HIF1Aa/b* and *HIF2Aa/b*. To compare the severity of hypoxic exposures across studies, we calculated the cumulative oxygen deficit (COD) for each sampling interval in each study (Nelson and Lipkey, 2015). COD was determined as the product of the duration of the hypoxic exposure (h) and the difference in DO concentration (mg L^-1^) between the air-saturated concentration and the reported concentration. The air-saturated DO concentration was determined for the temperature and salinity given in each study, assuming barometric pressure was 101.3 kPa. For example, in an experiment conducted in full strength sea water (salinity 36) at 25°C, when the air-saturated DO concentration is 6.73 mg L^-1^, a 4-h exposure at 1.0 mg L^-1^ corresponds to a COD of 23 h mg L^-1^, whereas a 7-day exposure at 2.4 mg L^-1^ corresponds to a COD of 727 h mg L^-1^. The change in *HIFA* mRNA abundance at every sampling interval was coded as a binary variable. When the authors reported a statistically significant increase in mRNA abundance compared to normoxia, this sampling interval was scored as “1”, whereas, sampling intervals showing no significant difference, or a significant decrease, were scored as “0”. Finally, each study’s qPCR methodology was evaluated for the Minimum Information for Publication of Quantitative Real-Time PCR Experiments (MIQE) (Bustin et al., 2009). We determined how many of the 34 criteria deemed to be essential when reporting qPCR experiments were stated in the original citations (Supplementary Data Sheet S2).

We used generalized linear mixed-effects models (Silk et al., 2020) to evaluate the influence of gene, tissue, COD, temperature, salinity, and MIQE reporting on whether *HIFA* mRNA abundance was reported to increase during hypoxia. Study was included as a random factor to allow for multiple, potentially non-independent, samples from a given study. We limited our analyses of the tissue-specificity of the hypoxia response in *HIFA* mRNA abundance to those tissues that had been reported in three or more studies because mixed effects models are less reliable at low values of the random factor (Silk et al., 2020). The resulting outputs reflect the predicted frequency of reporting an increase in *HIFA* mRNA (from 0 to 1) for a given fixed factor. We used R packages lme4 (Bates et al., 2015) and emmeans (Lenth et al., 2018) and R version 3.6.1 (R Studio Team, 2022).

## 3 Results and discussion

### 3.1 Description of dataset

The final dataset consisted of 38 studies reporting on *HIFA* mRNA abundance in 25 species of ray-finned fishes exposed to laboratory hypoxia (Table 1). Across all studies, there were 413 discrete sampling intervals, representing different fish species, *HIFA* genes, tissues sampled, and severity of hypoxia (DO level and duration). *HIFA* mRNA abundance was significantly higher in tissues of hypoxic fish compared to normoxic controls in 182 cases (44%), unchanged in 214 cases (52%), and significantly lower in 17 cases (4%).

**TABLE 1:** Summary of studies measuring *HIFA* mRNA by qPCR of tissues from adult ray-finned fishes exposed to laboratory hypoxia.

Species	Gene^a^	Temp (°C)	Salinity	DO (mg O_2_ L^-1^)	Duration (h)	Tissue	References
*Acipenser baerii*	1A, 2A, 3A	24.0	0.0	2.0	1, 3, 6, 12, 48, 96	Brain, gill, heart, liver	Wang et al. (2021b)
*Astronotus crassipinnis*	1Aa	27.0	0.0	0.5	3	Liver	Baptista et al. (2016)
*Astronotus crassipinnis*	1Aa	28.0	0.0	0.7	1, 3, 5	Liver, muscle	Heinrichs-Caldas et al. (2019)
*Callionymus valenciennei*	1Aa, 2Aa	20.5	32.5	1.40	48, 168	Liver	Kodama et al. (2012)
*Carassius auratus*	1Ab	21.0	0.0	2.6	168	Heart	Cameron et al. (2013)
*Catla catla*	1Ab	25.0	0.0	1.0, 3.0	1, 48	Brain, gill	Singh et al. (2016)
*Cirrhinus mrigala*	1Ab	28.0	0.0	0.5	1, 3, 12, 24, 72, 168, 360	Gill	Varghese et al. (2017)
*Cirrhinus mrigala*	1Ab	28.0	0.0	0.5	72	Gill	Varghese et al. (2022)
*Clarias batrachus*	1Ab, 2Aa	22.0	0.0	0.98	1, 6	Brain, liver, muscle	Mohindra et al. (2013)
*Clupea pallasii*	1Ab	12.8	35.0	2.3, 2.63, 4.25	0.5, 1, 2, 4, 8, 16	Liver	Froehlich et al. (2015)
*Danio rerio*	1Ab, 2Aa, 3A	24.0	0.0	2.5	1176	Intestine, liver, muscle	Ma et al. (2021)
*Dicentrarchus labrax*	1Aa	21.8	35.0	1.9, 4.3	4, 24, 48 120, 360	Liver	Terova et al. (2008)
*Fundulus grandis*	1Aa, 2Aa, 3A	24.6	9.0	1.0	6, 24	Gill, liver, muscle, ovary	Murphy et al. (2023)
*Gobionotothen gibberifrons*	1Aa	1.0	35.0	2.3, 5.0	2, 12	Heart	O’Brien et al. (2020)
*Larimichthys crocea*	1Aa	25.0	35.0	1.6	1, 3, 6, 12, 24	Brain	Liu et al. (2018a)
*Larimichthys crocea*	1Aa	22.0	29.0	2.0	1, 3, 6, 12, 24 48, 96	Liver	Luo et al. (2021)
*Larimichthys crocea*	1Aa	22.0	29.0	0.5, 2.5, 3.5, 4.5	3, 6, 12, 24 48, 72, 96	Gill, kidney, liver	Wang et al. (2017)
*Larimichthys crocea*	1Aa	23.6	25.7	1.5	3, 6, 12, 24, 48	Liver	Zeng et al. (2016)
*Larimichthys crocea*	1Aa	25.0	26.0	3.0	48	Liver	Zeng et al. (2020)
*Lepisosteus oculatus*	2A	22.0	0.0	3.6	1704	Gill, swim bladder	Rimoldi et al. (2016)
*Megalobrama amblycephala*	3A	22.0	0.0	1.0	4	Brain, kidney, liver	Liu et al. (2018b)
*Megalobrama amblycephala*	1Ab, 2Aa	22.0	0.0	1.0	4	Brain, kidney, liver	Shen et al. (2010)
*Megalobrama amblycephala*	1Ab	25.0	0.0	2.0	6, 12, 24	Gill	Yu et al. (2023)
*Micropogonias undulatus*	1Aa, 2Aa	27.0	32.0	1.7	12, 72, 168	Ovary	Rahman and Thomas (2007)
*Micropogonias undulatus*	1Aa	27.0	32.0	1.7	672	Liver	Rahman and Thomas (2011)
*Micropogonias undulatus*	2Aa	27.0	32.0	1.7	672	Liver	Rahman and Thomas (2012)
*Micropterus salmoides*	1Aa	11.0	0.0	1.2	1, 2, 4, 8, 12, 24	Brain, gill, liver	Yang et al. (2017)
*Notothenia coriiceps*	1Aa	1.0	35.0	2.3, 5.0	2, 12	Heart	O’Brien et al. (2020)
*Oreochromis niloticus*	1Aa	28.0	0.0	1.0	18	Liver	Abarike et al. (2020)
*Oreochromis niloticus*	1Aa	27.0	0.0	2.0	3, 8	Brain	Dourado et al. (2023)
*Oreochromis niloticus*	1Aa	26.5	0.0	0.7	6, 12, 24	Brain, gill, heart, liver	Li et al. (2017)
*Oreochromis niloticus*	1Aa	26.5	0.0	1.6	2, 4, 6, 8	Brain, liver	Li et al. (2019)
*Pelteobagrus fulvidraco*	1Ab, 2Aa	24.5	0.0	0.7	1.5, 4, 6.5	Brain, liver	Pei et al. (2021)
*Pelteobagrus fulvidraco*	1Ab, 2Aa	26.0	0.0	1.14	1, 3, 6	Brain, gill, liver	Wang et al. (2021a)
*Pelteobagrus vachelli*	1Ab, 2Aa, 3A	24.0	0.0	0.7	1.5, 4, 6.5	Liver	Zhang et al. (2017)
*Perca fluviatilis*	1Aa	19.0	0.0	0.4, 2.8	1,360	Brain, liver, muscle	Rimoldi et al. (2012)
*Rachycentron canadum*	1Aa	29.0	30.0	3.15	24, 168, 336, 672	Gill, intestine, liver, muscle	Huang et al. (2022)
*Schizothorax prenanti*	1Ab	17.0	0.0	1.2, 3.0	12, 24	Brain, gill, intestine, liver, muscle	Zhao et al. (2020)
*Sebastes schlegelii*	1Aa, 2Aa	24.0	30.0	4.5	1	Gill, liver, ovary, spleen	Mu et al. (2015)

^a^ Gene measured was determined by BLAST, searches against ray-finned fishes. Nomenclature follows Townley et al. (2022).

Thirty-four studies described the effects of hypoxic exposure on *HIF1A* mRNA levels, 13 described changes in *HIF2A* mRNA, and five described changes in *HIF3A* (Supplementary Figure S1A). All but two studies were on species from lineages arising after the teleost-specific genome duplication (Hughes et al., 2018), meaning that certain species were predicted to have teleost-specific “a” and “b” duplicates (Rytkonen et al., 2013; Townley et al., 2022). The exceptions were Wang X. et al. (2021), who reported on *HIF1A*, *HIF2A*, and *HIF3A* in Siberian sturgeon (*Acipenser baerii*), and Rimoldi et al. (2016), who reported on *HIF2A* from spotted gar (*Lepiosteus oculatus*).

Apart from these reports on basal taxa, 21 studies examined changes in *HIF1Aa* in Neoteleostei (more derived ray-finned fishes) and 13 examined changes in *HIF1Ab* in Otocephala. For *HIF2A*, all studies reported data for *HIF2Aa*, even though *HIF2Ab* is found in most fish lineages (Rytkonen et al., 2013; Townley et al., 2022). The lack of data on *HIF2Ab* is a critical gap in knowledge, considering that recent work suggests that sequence variation in this duplicate is associated with variation in hypoxia tolerance among ray-finned fishes (Babin et al., 2024). For *HIF3A*, only a single teleost-specific duplicate has been retained in ray-finned fishes (Townley et al., 2022), and we did not distinguish between “a” and “b” duplicates. No studies were found on *HIF4A*.

### 3.2 Effects of gene and teleost-specific duplicate

Prior to assessing differences in *HIF1A*, *HIF2A*, and *HIF3A*, we compared the frequency of reported hypoxia-dependent increases in mRNA abundance for *HIF1Aa* and *HIF1Ab* (excluding data for ancestral *HIF1A* from Siberian sturgeon). No significant difference was found in the frequency of reported increases in the levels of *HIF1Aa* and *HIF1Ab* mRNA (*p* = 0.160) (Supplementary Table S1). Thus, data were combined for teleost-specific duplicates, along with ancestral forms, of each gene. Hereafter, *HIF1A*, *HIF2A*, and *HIF3A* refer to data from all forms of each gene.

**FIGURE 1:**
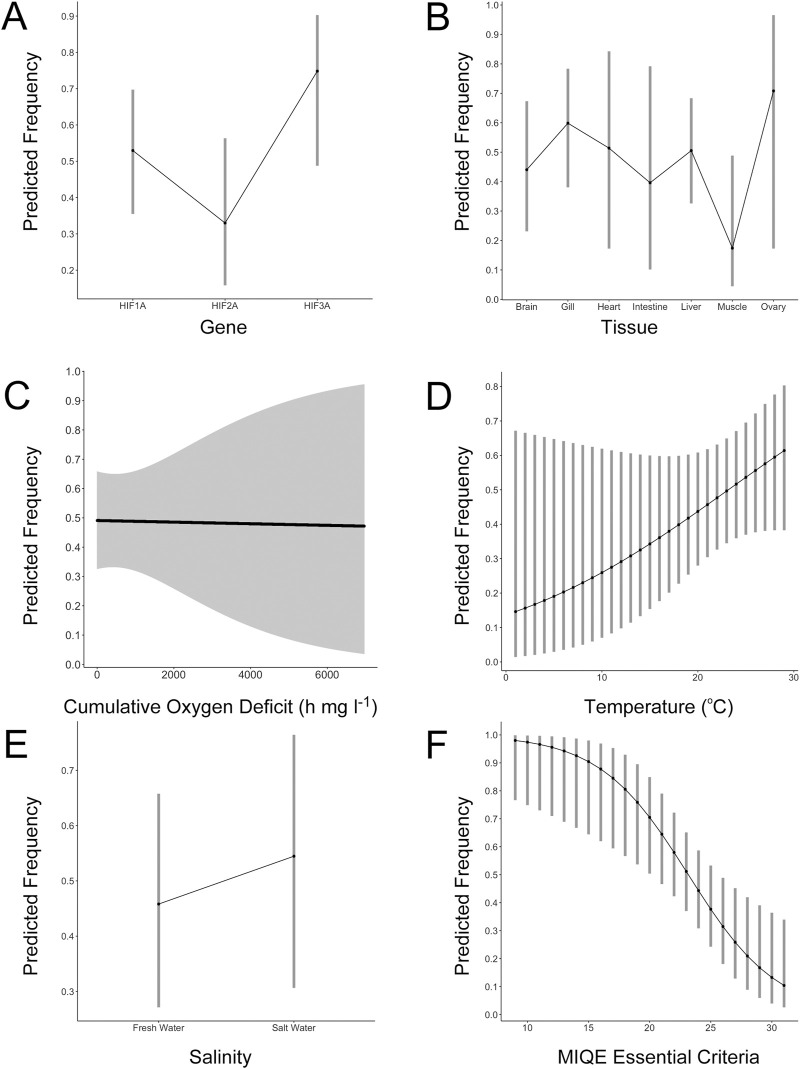
Results of generalized linear mixed effects modeling of factors influencing the abundance of *HIFA* mRNA during hypoxic exposure of adult ray-finned fishes. **(A)** The effects of gene (*HIF1A*, *HIF2A*, and *HIF3A*) on the predicted frequency of increased *HIFA* mRNA abundance. **(B)** The effects of tissue on the predicted frequency of increased *HIF1A* mRNA abundance. **(C)** The effects of cumulative oxygen deficit on the predicted frequency of increased *HIF1A* mRNA abundance. **(D)** The effects of experimental temperature on the predicted frequency of increased *HIF1A* mRNA abundance. **(E)** The effects of experimental salinity on the predicted frequency of increased *HIF1A* mRNA abundance. **(F)** The effects of methods reporting (number of MIQE essential criteria) on the predicted frequency of increased *HIF1A* mRNA abundance. For all panels, the *y*-axis represents the model-predicted frequency of increased *HIFA* mRNA abundance during hypoxic exposure and the error bars represent the 95% confidence intervals of the estimates. See Supplementary Tables S2–S5 for complete model results.

The effect of *HIFA* gene on the frequency of reported hypoxia-induced increases in mRNA abundance approached statistical significance (Supplementary Table S2). The frequency of increased *HIF1A* mRNA tended to be higher than that for *HIF2A* (*p* = 0.0525), but lower than that for *HIF3A* (*p* = 0.0566) (Figure 1A). Law et al. (2006) used Northern blot analysis and found higher *HIF3A* mRNA levels in several tissues of the grass carp (*Ctenopharyngodon idella*) under hypoxia when *HIF1A* mRNA levels were largely unaffected in the same tissues. These observations, combined with the widespread expression of *HIF3A* in tissues of normoxic fish (Townley et al., 2022), suggest that more attention should be given to this understudied gene (Duan, 2016).

### 3.3 Effects of tissue

Measurements of mRNA abundance were not uniformly distributed among tissues (Supplementary Figure S1B). Across all *HIFA* genes, the most well-documented tissue was liver, followed by gill, brain, skeletal muscle, heart, kidney, ovary, intestine, spleen, testes, and swim bladder. For *HIF1A*, the frequency of hypoxia-induced increases in mRNA was highly variable and not significantly different across tissues (Figure 1B; Supplementary Table S3), although there tended to be fewer reports of increased *HIF1A* mRNA in skeletal muscle (*p* = 0.113). For *HIF2A*, there was a trend toward a higher frequency of reported effects of hypoxia in gill (*p* = 0.0782; Supplementary Table S4). Interestingly, *HIF2A* is more highly expressed in gill than in other tissues even under normoxia (Townley et al., 2022) and it may play a role in oxygen-sensing in fishes (Pan et al., 2022). For *HIF3A*, only one tissue (liver) was measured in at least three studies, precluding analysis of the effects of tissue on mRNA changes during hypoxia.

### 3.4 Effects of exposure conditions

We evaluated the effects of exposure conditions (COD, temperature, and salinity) on the frequency of reported increases in *HIF1A* mRNA, pooling measurements from all tissues (Supplementary Table S5). Surprisingly, COD, which reflects both the duration and the depth of hypoxia (Nelson and Lipkey, 2015), had no effect on *HIF1A* abundance (*p* = 0.918; Figure 1C). The only pattern was that the variability in responses increased as a function of COD. Because we pooled all tissues for this analysis, it is possible that tissue-specific responses were obscured (e.g., increasing mRNA levels over time *versus* decreasing over time in different tissues). Consequently, we repeated the analysis on the two most-studied tissues, liver and gill; COD was not statistically related to the frequency of increased abundance of liver *HIF1A* (*p* = 0.786) or gill *HIF1A* (*p* = 0.472). For liver *HIF1A*, some studies reported higher mRNA levels with increasing time of exposure, as one might expect (Froehlich et al., 2015; Heinrichs-Caldas et al., 2019). However, there were more studies where *HIF1A* was not different from controls at any time point (Li et al., 2017; Murphy et al., 2023; Wang X. et al., 2021; Yang et al., 2017) or it was higher at all time points (Huang et al., 2022; Luo et al., 2021). Thus, there was no consistent pattern across all studies due to the duration or severity of hypoxia.

We also evaluated temperature and salinity as potential factors related to the increase in *HIFA* mRNA abundance. We observed a non-significant trend toward a greater frequency of increased *HIF1A* mRNA at increasing temperature (*p* = 0.114; Figure 1D). Using Northern blot analyses, Rissanen et al. (2006) presented evidence of an interaction between temperature and hypoxia exposure on levels of *HIF1A* mRNA in Crucian carp, although this effect differed among tissues. When data from freshwater species were grouped and compared to data pooled from estuarine and marine species, there was no effect of salinity on the frequency of elevated *HIF1A* mRNA (*p* = 0.394; Figure 1E). Like the effects of temperature, though, direct tests of the effects of salinity on *HIFA* mRNA abundance in single study are scarce. COD, temperature, and salinity were unrelated to the frequency of reported increases in *HIF2A* or *HIF3A* mRNA (Supplementary Tables S6, S7; Supplementary Figure S2).

### 3.5 Effects of methods reporting

We determined whether the number of MIQE criteria deemed essential for ensuring data uniformity, comparability, and reliability (Bustin et al., 2009) reported in each study was related to the frequency of elevated mRNA abundance during hypoxia (pooling tissues and sampling intervals for a given *HIFA* transcript). For *HIF1A*, there was a strong, non-linear, negative relationship between the number of essential MIQE criteria and the frequency of reported increases in mRNA abundance (*p* = 0.0032; Figure 1F; Supplementary Table S8). Studies that reported more details were less likely to find increased *HIF1A* mRNA compared to those reporting fewer of these details. Similar results were generated for *HIF2A* (*p* = 0.127; Supplementary Figure S3A; Supplementary Table S9) and HIF3A (*p* = 0.0547; Supplementary Figure S3B; Supplementary Table S10), although they were not individually significant due to a much smaller number of observations.

Some of the MIQE criteria that were under-reported in the literature include assessment of RNA integrity prior to reverse transcription, linearity of calibration curves, and justification of reference genes. The last is especially important because many of the “housekeeping” genes used to standardize the levels of a gene of interest can be influenced by hypoxia (e.g., GAPDH). If the abundance of a reference gene is lower during hypoxia, then levels of *HIFA* mRNA will be overestimated. In addition, given the above-mentioned diversity of *HIFA* paralogs among fishes, the targets of pPCR must be unambiguously identified. Unfortunately, no consensus exists on the nomenclature of teleost-specific duplicates of *HIFA*, which has led to the different names for a given gene (e.g., *HIF2Aa*) or uninformative names (e.g., *HIF1-like*). We adhered to the nomenclature of Townley et al. (2022) and Babin et al. (2024), which are the most complete phylogenetic surveys of *HIFA* in ray-finned fishes at the present time.

### 3.6 Limitations of this study

One limitation of this study is that we cannot exclude the possibility that the response of *HIFA* mRNA to hypoxia is species-specific. We included study as a random factor in our analyses, which would largely explain the same variation as species because all but one study (O’Brien et al., 2020) measured a single species. Nevertheless, the lack of consistent responses across studies, species, tissues, and experimental conditions suggests that attempts to use *HIFA* mRNA as a biomarker of hypoxic exposure (e.g., Zhang et al., 2009; Thomas and Rahman, 2009; Froehlich et al., 2015) must be coupled with careful laboratory experiments that adhere to guidelines for conducting and reporting qPCR. Moreover, those results might be useful only for that species. A second limitation is that our analyses considered only author-reported significant differences, rather than the magnitude of the changes in *HIFA* mRNA abundance. Considering the great variability in the magnitude of those changes across studies, however, it is unlikely that including effect sizes in our analyses would have led to dramatically different conclusions. Finally, we calculated the cumulative oxygen deficit for each sampling interval to represent the degree of hypoxic exposure. Because fish species are known to differ in their hypoxia tolerance (Verberk et al., 2022), relating the DO level of the laboratory exposures to some measure of each species’ hypoxia tolerance, for example, the critical oxygen tension (Ultsch and Regan, 2019), could be a better metric of the stress experienced. Unfortunately, there is not a single measure of hypoxia tolerance that is universally accepted (Wood, 2018) or available for all the species represented in the current analysis.

## 4 Conclusion

Based upon our results, we conclude that increased *HIFA* mRNA during hypoxia is not a universal response of fish to low oxygen, but one that appears to be variable among studies and best explained by the rigor of reporting experimental methods rather than any other factor. Even allowing for species-specific increases in *HIFA* mRNA, several important questions remain. First, what is the mechanism of this increase? To address this question, studies should focus on the upstream regulators of *HIFA* transcription, including elucidating specific transcription factor binding sites in the promoter of *HIFA* genes (e.g., Rytkonen et al., 2013). For example, is *HIFA* transcription under the control of HIF, itself, or some other transcription factor? Second, does an increase in *HIFA* mRNA result in an increase in the corresponding HIFα protein? The abundance of mRNA does not always correlate with protein abundance due to post-transcriptional regulation, including changes in protein stability (Vogel and Marcotte, 2012). Early studies in fish cell culture (Soitamo et al., 2001) and recent studies on tissues of fish exposed to hypoxia (Murphy et al., 2023) showed that HIFα protein levels increase even when *HIFA* mRNA does not. Clearly, more studies need to couple measures of *HIFA* mRNA with measures of HIFα protein. Third, and most importantly, do changes in *HIFA* mRNA result in changes in gene expression and phenotypes that improve the fish’s capacity to tolerate aquatic hypoxia? With the advent of broad scale measures of mRNA abundance, it is possible to correlate levels of *HIFA* mRNA (or HIFα protein) and levels of target genes or pathways. Experiments such as these would enhance our understanding of HIF’s role in the responses of fish to aquatic hypoxia.

## Supplementary material

SUPPLEMENTARY TABLE 1 Tables S1-S10.

SUPPLEMENTARY IMAGE 1 Figures S1-S3.
