# Supplementary material for: Diverse responses of hypoxia-inducible factor alpha mRNA abundance in fish exposed to low oxygen: the importance of reporting methods
Source: Front Physiol. 2024 Oct 4;15:1496226. doi: 10.3389/fphys.2024.1496226 (PMC11486919; doi:10.3389/fphys.2024.1496226)
Supplement: Supplementary file 1 [file Table1.DOCX]

**Table S1:** Results of generalized linear mixed effects model analysis of the influence of teleost-specific duplicate on the frequency of reported increases in *HIF1A* mRNA. Estimates are on the logit scale and the effect of *HIF1Ab* is expressed relative to *HIF1Aa* (Intercept).

|  | Estimate | SE | z | P |
| --- | --- | --- | --- | --- |
| Intercept | -0.3617 | 0.4382 | -0.825 | 0.409 |
| *HIF1Ab* | 1.0058 | 0.7153 | 1.406 | 0.160 |

**Table S2:** Results of generalized linear mixed effects model analysis of the influence of gene on the frequency of reported increases in *HIFA* mRNA. Estimates are on the logit scale and the effects of *HIF2A* and *HIF3A* are expressed relative *HIF1A* (Intercept).

|  | Estimate | SE | z | P |
| --- | --- | --- | --- | --- |
| Intercept | 0.1183 | 0.3653 | 0.324 | 0.7461 |
| *HIF2A* | -0.8271 | 0.4266 | -1.939 | 0.0525 |
| *HIF3A* | 0.9717 | 0.5096 | 1.907 | 0.0566 |

**Table S3:** Results of generalized linear mixed effects model analysis of the influence of tissue on the frequency of reported increases in *HIF1A* mRNA. Estimates are on the logit scale and the effects of all tissues are expressed relative to brain (Intercept).

|  | Estimate | SE | z | P |
| --- | --- | --- | --- | --- |
| Intercept | -0.2394 | 0.4916 | -0.487 | 0.626 |
| Gill | 0.6390 | 0.5329 | 1.199 | 0.230 |
| Heart | 0.2948 | 0.8699 | 0.339 | 0.735 |
| Intestine | -0.1823 | 0.9455 | 0.193 | 0.847 |
| Liver | 0.2609 | 0.5002 | 0.522 | 0.602 |
| Muscle | -1.3175 | 0.8320 | -1.583 | 0.113 |
| Ovary | 1.1260 | 1.3153 | 0.856 | 0.392 |

**Table S4:** Results of generalized linear mixed effects model analysis of the influence of tissue on the frequency of reported increases in *HIF2A* mRNA. Estimates are on the logit scale and the effects of all tissues are expressed relative to brain (Intercept).

|  | Estimate | SE | z | P |
| --- | --- | --- | --- | --- |
| Intercept | -2.0179 | 1.3229 | -1.525 | 0.1272 |
| Gill | 3.1167 | 1.7698 | 1.761 | 0.0782 |
| Liver | 1.3825 | 1.1304 | 1.223 | 0.2213 |
| Muscle | -18.1510 | 1024 | -0.018 | 0.9859 |
| Ovary | -0.1303 | 2.4475 | -0.053 | 0.9576 |

**Table S5:** Results of generalized linear mixed effects model analysis of the influence of experimental conditions (cumulative oxygen deficit, temperature, and salinity) on the frequency of reported increases in *HIF1A* mRNA. Estimates are on the logit scale. The effects of COD and temperature are given as slopes and evaluated against no effect (slope = 0). The effect of salinity is for experiments conducted in brackish or sea water compared to fresh water.

|  | Estimate | SE | z | P |
| --- | --- | --- | --- | --- |
| Intercept | -2.236 | 1.355 | -1.650 | 0.099 |
| COD | -0.00002401 | 0.0002345 | -0.102 | 0.918 |
| Temp | 0.08753 | 0.05544 | 1.579 | 0.114 |
| Salinity | 0.5489 | 0.6437 | 0.853 | 0.394 |

**Table S6:** Results of generalized linear mixed effects model analysis of the influence of experimental conditions (cumulative oxygen deficit, temperature, and salinity) on the frequency of reported increases in *HIF2A* mRNA. Estimates are on the logit scale. The effects of COD and temperature are given as slopes and evaluated against no effect (slope = 0). The effect of salinity is for experiments conducted in brackish or sea water compared to fresh water.

|  | Estimate | SE | z | P |
| --- | --- | --- | --- | --- |
| Intercept | -8.69799 | 11.52912 | -0.754 | 0.451 |
| COD | 0.0004028 | 0.0003863 | 1.045 | 0.296 |
| Temperature | 0.30246 | 0.47825 | 0.632 | 0.527 |
| Salinity | 0.00197 | 1.87186 | 0.001 | 0.999 |

**Table S7:** Results of generalized linear mixed effects model analysis of the influence of cumulative oxygen deficit on the frequency of reported increases in *HIF3A* mRNA. The effect of COD is given as a slope and evaluated against no effect (slope = 0). Estimates are on the logit scale. There was insufficient replication to assess the effects of experimental temperature and salinity.

|  | Estimate | SE | z | P |
| --- | --- | --- | --- | --- |
| Intercept | -0.223209 | 0.9455463 | -0.236 | 0.813 |
| COD | -0.0001040 | 0.0003244 | -0.321 | 0.749 |

**Table S8:** Results of generalized linear mixed effects model analysis of the influence of methods reporting (the number of essential MIQE criteria) on the frequency of reported increases in *HIF1A* mRNA. This analysis pooled all data from *HIF1A* (all tissues and experimental conditions). Estimates are on the logit scale. The effect of MIQE criteria is given as a slope and evaluated against no effect (slope = 0).

|  | Estimate | SE | z | P |
| --- | --- | --- | --- | --- |
| Intercept | 6.37378 | 2.21060 | 2.883 | 0.00394 |
| MIQE Criteria | -0.27510 | 0.09331 | -2.948 | 0.00320 |

**Table S9:** Results of generalized linear mixed effects model analysis of the influence of methods reporting (the number of essential MIQE criteria) on the frequency of reported increases in *HIF2A* mRNA. This analysis pooled all data from *HIF2A* (all tissues and experimental conditions). Estimates are on the logit scale. The effect of MIQE criteria is given as a slope and evaluated against no effect (slope = 0).

|  | Estimate | SE | z | P |
| --- | --- | --- | --- | --- |
| Intercept | 12.1061 | 8.5540 | 1.415 | 0.157 |
| MIQE Criteria | -0.5498 | 0.3602 | -1.526 | 0.127 |

**Table S10:** Results of generalized linear mixed effects model analysis of the influence of methods reporting (the number of essential MIQE criteria) on the frequency of reported increases in *HIF3A* mRNA. This analysis pooled all data from *HIF3A* (all tissues and experimental conditions). Estimates are on the logit scale. The effect of MIQE criteria is given as slope and evaluated against no effect (slope = 0).

|  | Estimate | SE | z | P |
| --- | --- | --- | --- | --- |
| Intercept | 20.1387 | 10.5804 | 1.903 | 0.0570 |
| MIQE Criteria | -0.8868 | 0.4616 | -1.921 | 0.0547 |
