# Supplementary material for: Diverse responses of hypoxia-inducible factor alpha mRNA abundance in fish exposed to low oxygen: the importance of reporting methods
Source: Front Physiol. 2024 Oct 4;15:1496226. doi: 10.3389/fphys.2024.1496226 (PMC11486919; doi:10.3389/fphys.2024.1496226)

Figure S1. Summary of **(A)** *HIFA* genes and **(B)** tissues sampled in studies reviewed here. In each panel, the wedges represent the proportion of studies examining each *HIFA* gene or tissue. The number of studies and the number of discrete sampling points are shown in parentheses (studies; samples). For panel **(A)**, all forms of each gene are combined (ancestral and teleost-specific duplicates). For panel **(B)**, "Other" represents one sample each from spleen, swim bladder, and testis.

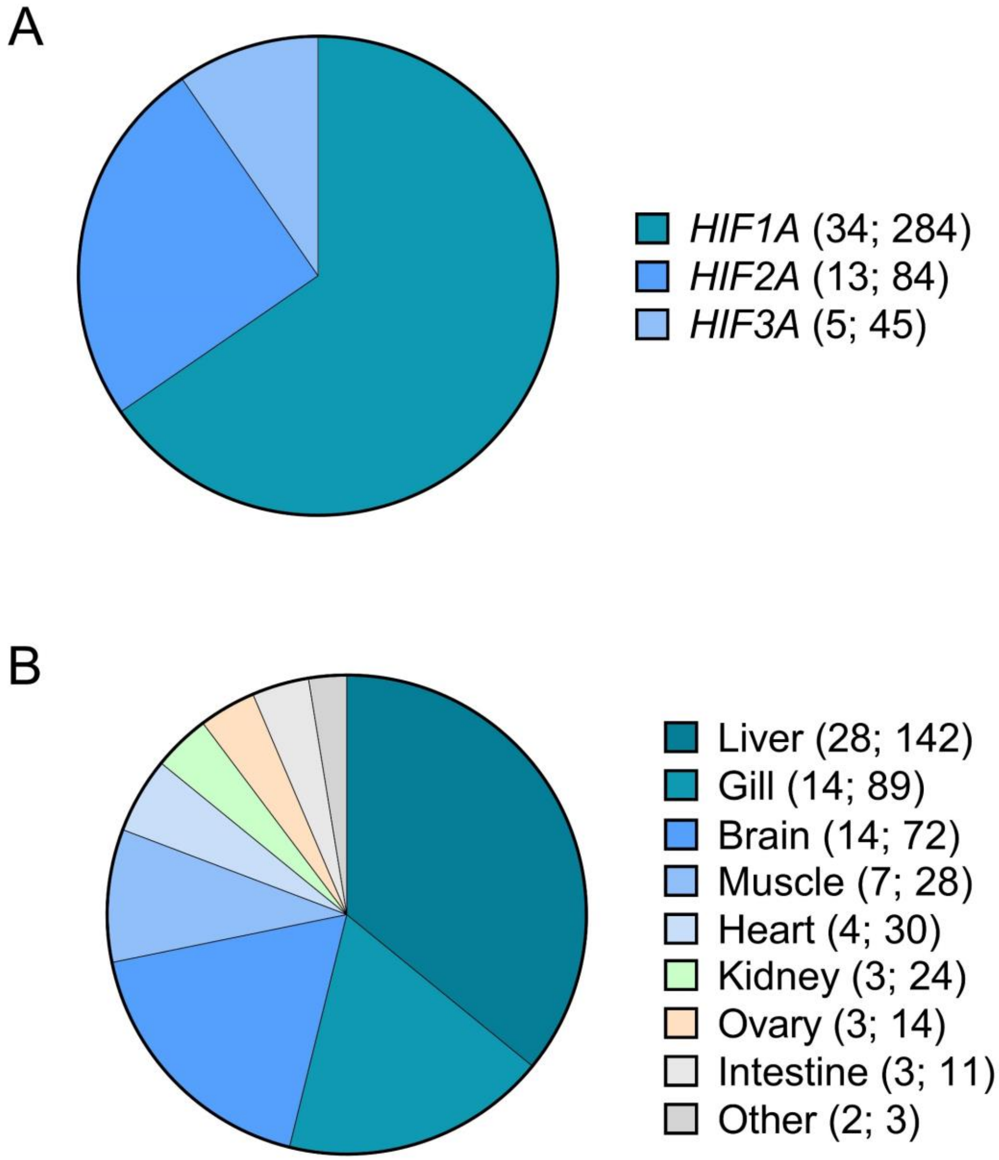

Figure S2. Results of generalized linear mixed effect model analysis of the effects of cumulative oxygen deficit on the predicted frequency of **(A)** *HIF2A* and **(B)** *HIF3A* mRNA increases during hypoxic exposure of fishes. The effect of COD was not significant for *HIF2A* (P = 0.296) or *HIF3A* (P = 0.749). See Tables S6 and S7 for complete statistical outputs.

A

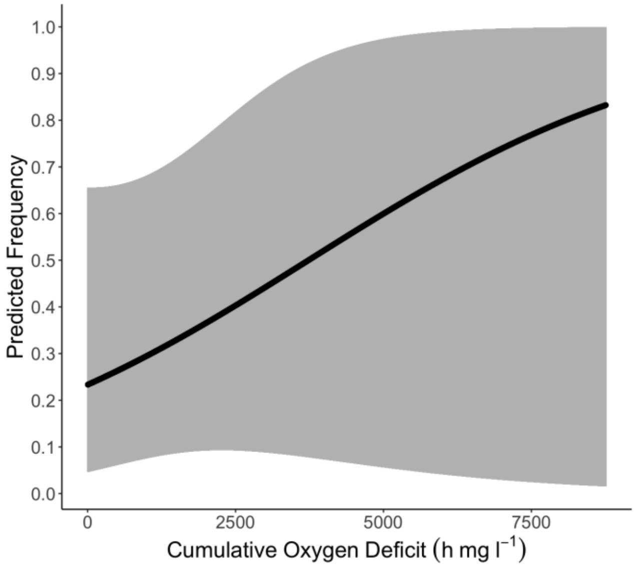

B

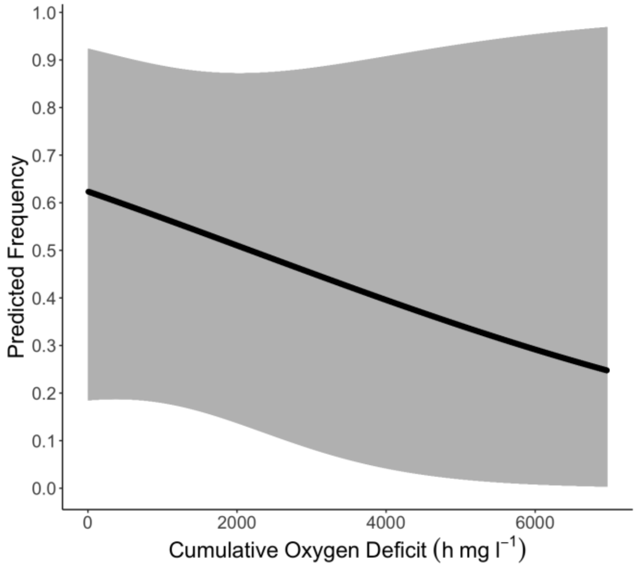

Figure S3. Results of generalized linear mixed effect model analysis of the effects of methods reporting on the predicted frequency of **(A)** *HIF2A* and **(B)** *HIF3A* mRNA increases during hypoxic exposure of fishes. Models included the number of essential MIQE criteria as a fixed effect and study as a random effect. The effect of MIQE score was not significant for *HIF2A* (P = 0.127) but it approached statistical significance for *HIF3A* (P = 0.0547). See Tables S9 and S10 for complete statistical outputs.

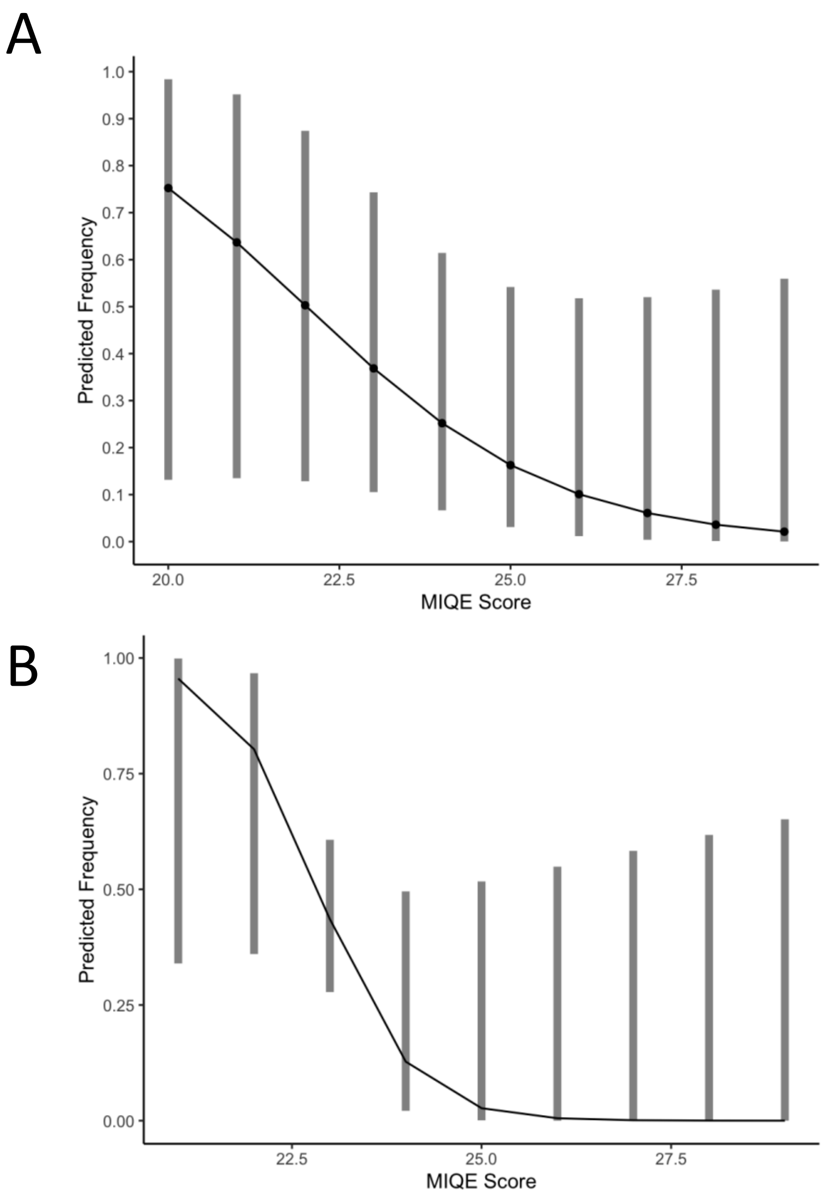

Supplement: Supplementary file 4 [file Image1.PDF]
